# Supplementary material for: Cocktails of NSAIDs and 17α Ethinylestradiol at Environmentally Relevant Doses in Drinking Water Alter Puberty Onset in Mice Intergenerationally
Source: Int J Mol Sci. 2023 Mar 20;24(6):5890. doi: 10.3390/ijms24065890 (PMC10099742; doi:10.3390/ijms24065890)
Supplement: Supplementary file 1 [file ijms-24-05890-s001.zip › ijms-2230313-SI.pdf]

Supp Figure S1

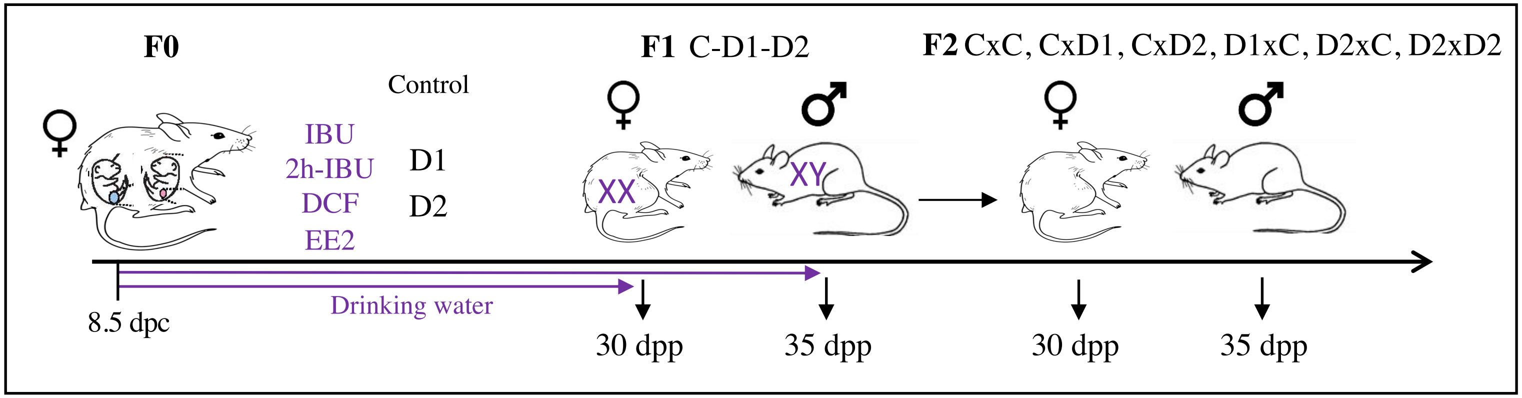

Supplementary Figure S1. Experimental design: the exposure to two mixtures (Dose 1 (D1) and Dose 2 (D2)) containing ibuprofen (IBU)/ 2hydroxyIbuprofen (2hIBU), diclofenac (DCF) and 17α-ethinyl-estradiol (EE2) was carried out in drinking water of pregnant mice, from 8.5 dpc (days post coitum) until sacrifice (30 dpp for the females; 35 dpp for the males) (dpp: days postpartum) to generate F1 D1 and F1 D2 ; the control (C) animals are generated after exposure to 0.001% ethanol. F1 male (m) or female (f) animals were mated with control male (m) or female (f) animals (n=6 groups: mCtrlxfCtrl (CxC), mCtrlxfD1 (CxD1), mCtrlxfD2 (CxD2), mD1xfCtrl, (D1xC), mD2xfCtrl (D2xC)) and mD2xfD2 (D2xD2)).

Supp Figure S2

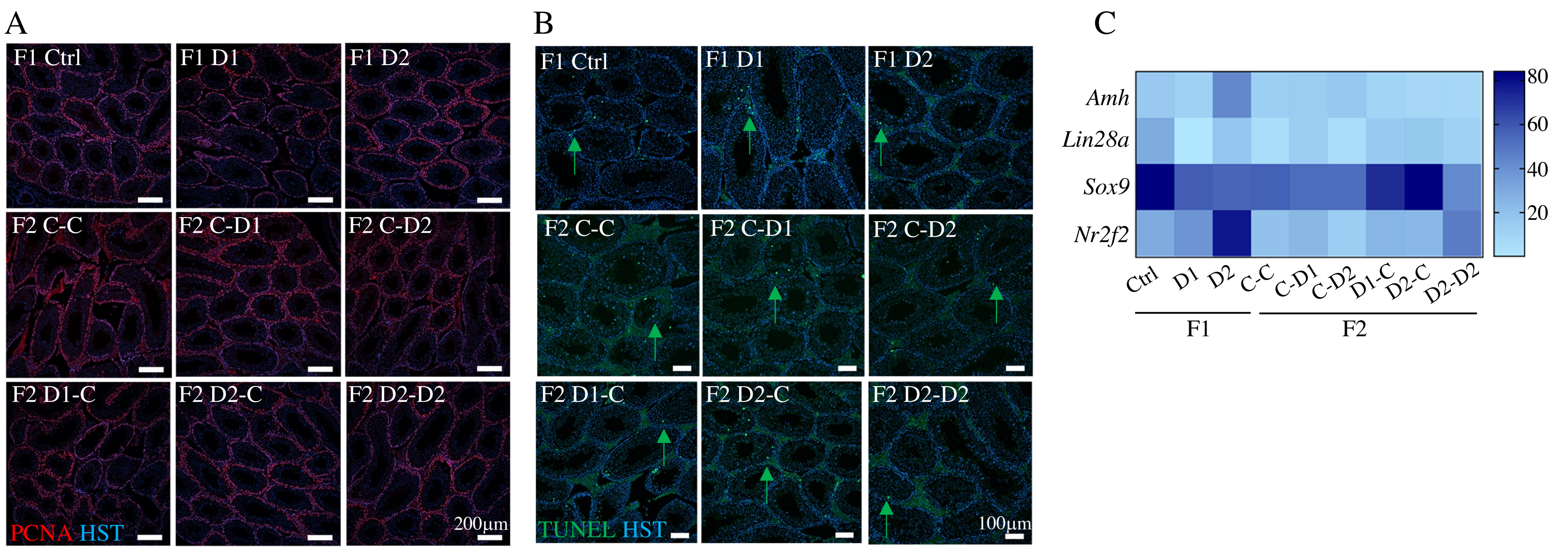

Supplementary Figure S2. Immunofluorescence staining of 35 dpp F1 and F2 testes. Paraffin-embedded testis tissue sections were used to assess (**A**) PCNA expression (cell proliferation marker; red). Scale bars: 200µm; and (**B**) cell apoptosis (TUNEL assay). Green arrows: apoptotic cells. Scale bars: 100µm. Nuclei were counterstained with Hoechst (HST, in blue). (**C**) Heatmap of DEGs in F1 and F2 males: testicular genes, *Amh, Lin28a, Sox9 and Nr2f2*.

Supp Figure S3

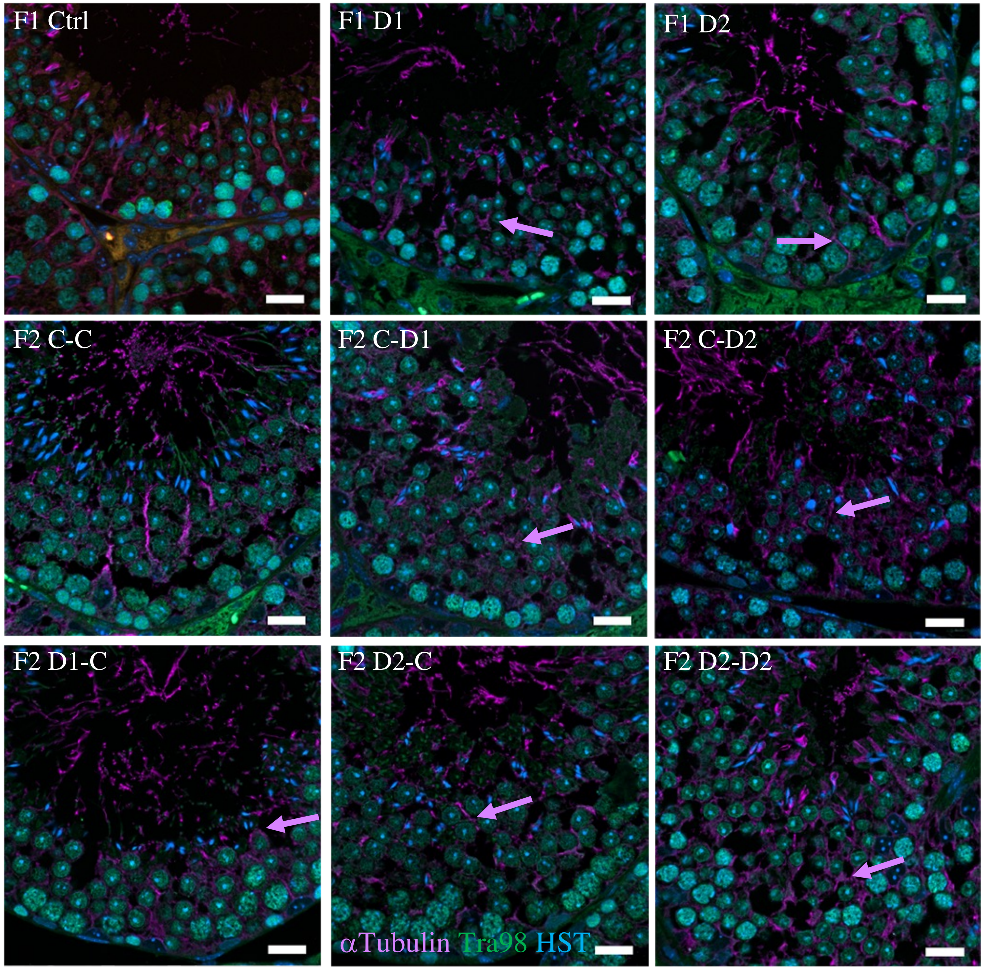

Supplementary Figure S3. Immunofluorescence staining of 35 dpp F1 and F2 testes (stage IV-VII tubules). Paraffin-embedded testis tissue sections were incubated with antibodies against a-tubulin (purple) and TRA98 (germ cells; green). Scale bars: 100µm. Nuclei were counterstained with Hoechst (HST; blue).

Supp Figure S4

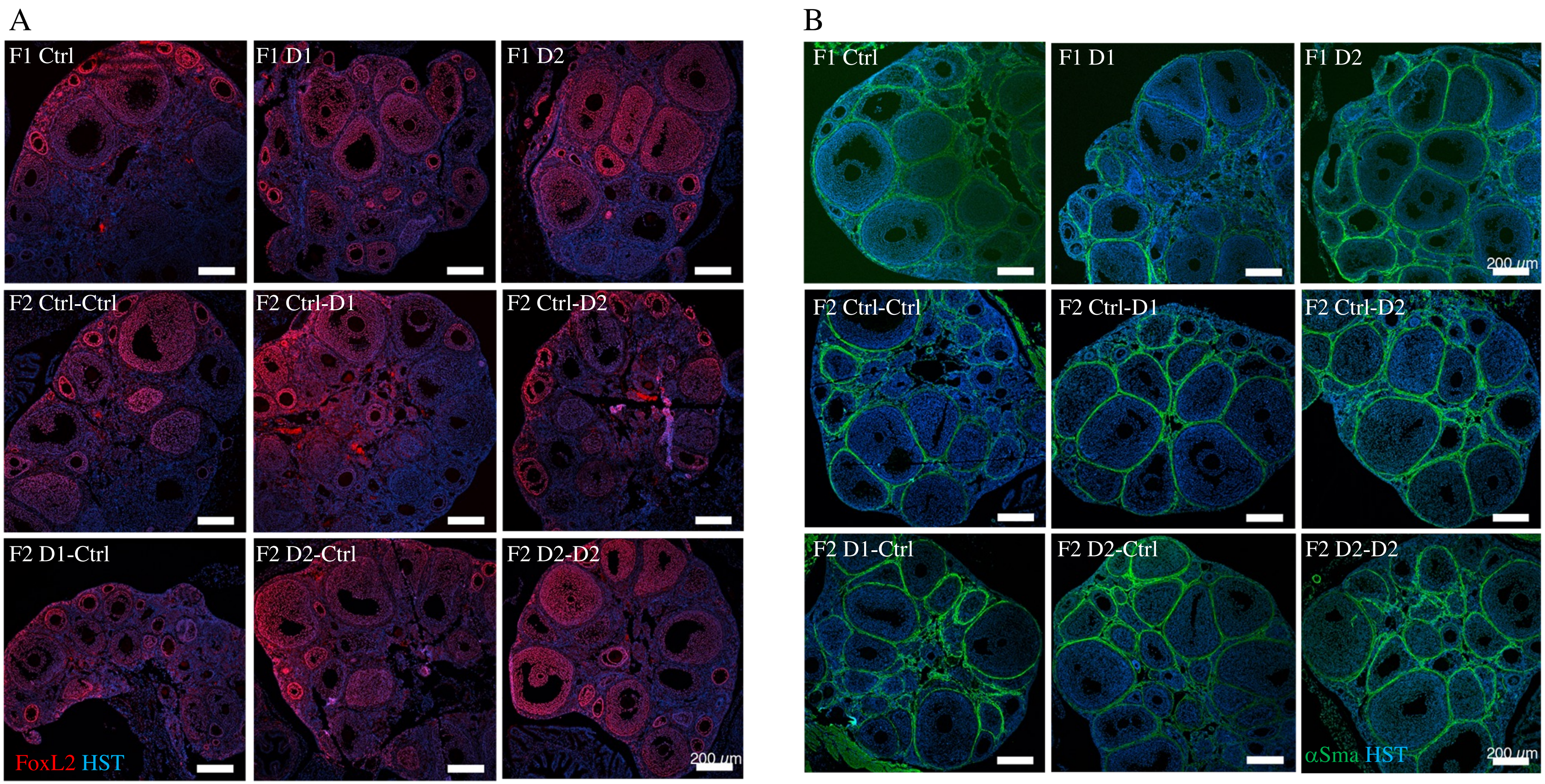

Supplementary Figure S4. Immunofluorescence staining of 30 dpp F1 and F2 ovaries. Paraffin-embedded ovary tissue sections were incubated with antibodies against (A) FOXL2 (granulosa cell marker; red) and (B) alpha-smooth muscle actin (a-SMA) to delineate ovarian follicles (green). Nuclei were counterstained with Hoechst (HST; blue). Scale bars: 200mm.

Supp Figure S5

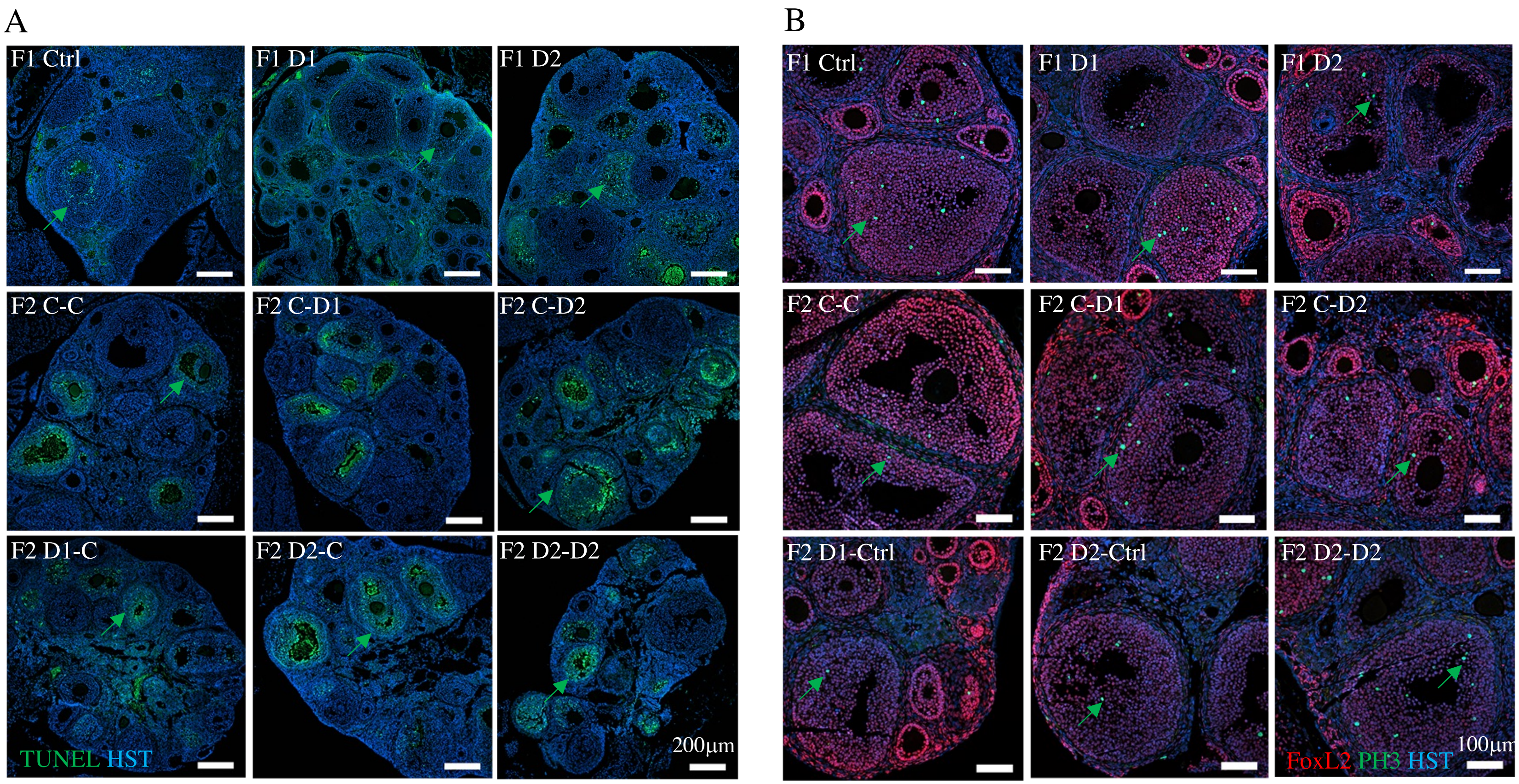

Supplementary Figure S5. Immunofluorescence staining of 30 dpp F1 and F2 ovaries. Paraffin-embedded ovary sections were stained with (**A**) TUNEL for apoptosis analysis; scale bars: 200mm. Green arrows: apoptotic follicles; and (**B**) PH3 (cell proliferation, S-phase, marker; red; scale bars: 100mm. Green arrows: proliferating follicular cells. Nuclei were counterstained with Hoechst (HST; blue).

Supp Figure S6

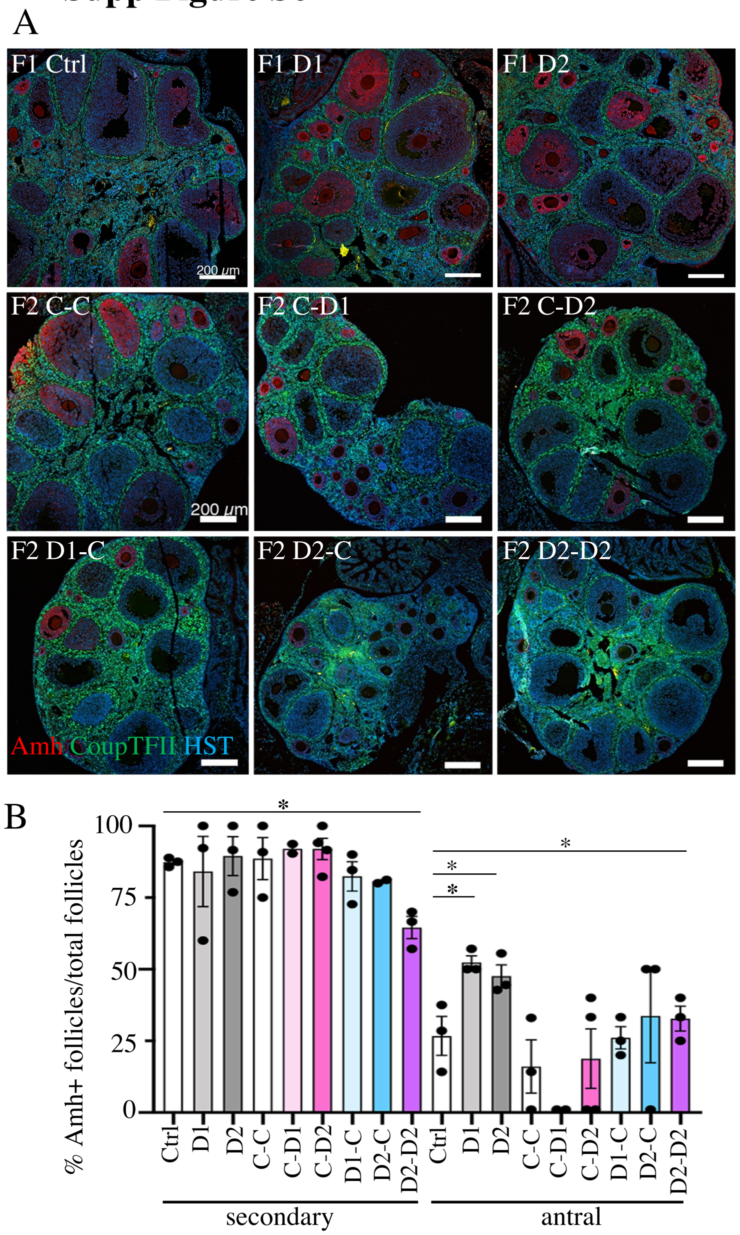

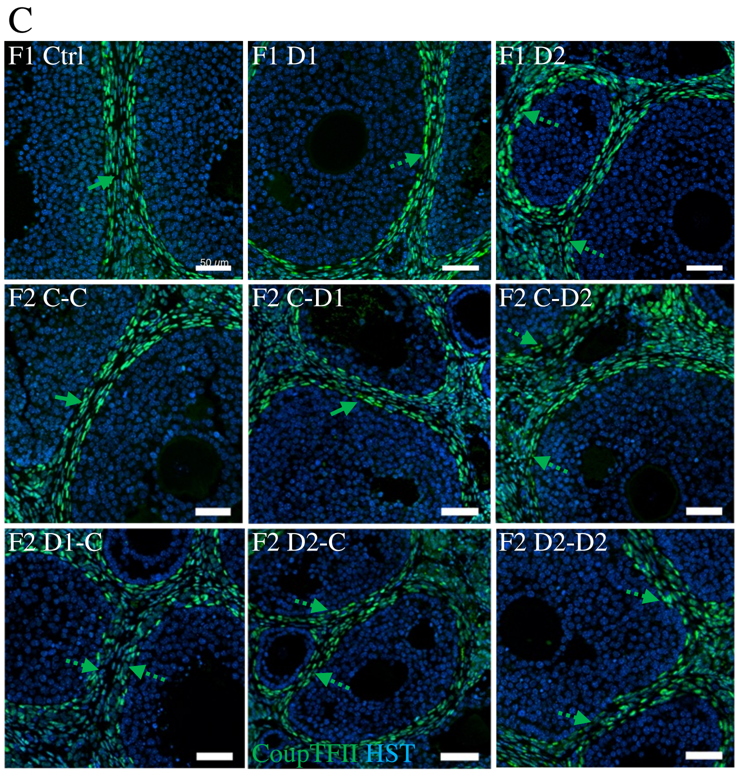

Supplementary Figure S6. Immunofluorescence staining of 30 dpp F1 and F2 ovaries. Paraffin-embedded ovary tissue sections were incubated with antibodies against (**A**) AMH (red) and COUPTFII (N2rf2; green). Scale bars: 200μm, and (**C**) COUPTFII (N2rf2; green) at higher magnification to highlight abnormal theca cell layers (dashed green arrows), scale bars: 50μm. Nuclei were counterstained with Hoechst (HST; blue). (**B**) Percentage of AMH-positive secondary or antral follicles among total number of follicles (represented as means +/- SEM (n= 2-3 ovaries/group)).
